# A Novel Radiotherapy Approach for Keloids with Intrabeam

**DOI:** 10.1155/2019/4693528

**Published:** 2019-07-22

**Authors:** Xiaojing Yang, Yuhui Shao, Weiwei Yu, Xiulong Zhang, Yi Sun, Lihua Zhang, Hongling Li, Xinmiao Yang, Jie Fu

**Affiliations:** Department of Radiation Oncology, Shanghai Jiao Tong University Affiliated Sixth People's Hospital, No. 600, Yishan Road, Shanghai, 200233, China

## Abstract

**Background:**

Keloids are hard nodules or plaques formed by excessive proliferation of connective tissue. Radiotherapy, widely used in various benign and malignant skin diseases, is an effective treatment for keloids. This work evaluates Intrabeam photon radiotherapy in the management of keloids.

**Methods:**

Fourteen patients who have undergone Intrabeam radiotherapy for a total of 15 sites of keloids were followed up. Twelve cases were first onset and the other two had recurrent diseases. Thirteen patients underwent surgical resection of keloids before radiotherapy. One relapsing patient received only 2 rounds of radiation therapy as she could not be reoperated. Radiotherapy was divided into 2 sessions on days 0 and 3 after surgery. The dose was 4 or 5 Gy each time for 3 min 14 s to 12 min 1 s. In addition, we compared our data to the recurrence of keloids in fourteen patients who had previously been exposed to electron beam using conventional accelerators.

**Results:**

We analyzed the treatment for adverse reactions and recurrence. In the Intrabeam group, one patient developed superficial skin ulcers a month after treatment. No one experienced wound rupture, bleeding, infection, skin contractures, or obvious hyperpigmentation. None of the fourteen cases showed any recurrence so far after on median 22.5 months of follow-up. Five patients in the electron beam group relapsed 3 to 10 months after treatment.

**Conclusion:**

Here, Intrabeam photon radiotherapy was shown to be an effective treatment for keloid scars and it is therefore recommended for management of this disease.

## 1. Introduction

Keloids, hard masses on the surface of the skin, are difficult to handle and prone to expansion and recurrence [1]. They are also accompanied by itching and are disturbing for the patients [2]. Although the years of clinical practice and numerous research studies have made great achievements in understanding the mechanisms, preventing, and treating keloids, there are still no satisfactory, particularly effective prevention and control strategies. The main methods for treating the disease include local injection of glucocorticoids, surgical resection, cryotherapy, radiation therapy, and compression therapy [3, 4]. However, the problem of recurrence of keloids still cannot be completely solved, and it has become a difficult problem for doctors and patients.

Since its discovery, X-ray radiation has been widely used in the treatment of skin diseases. Radiation therapy uses radiation to irradiate tissue, generate secondary electrons in the body, and cause ionization, which inhibits cell division and proliferation by directly or indirectly impacting DNA strand and breaking its molecular chains [5]. Rapidly dividing and proliferating cells are sensitive to radiotherapy. Radiotherapy is considered one of the most effective treatments for keloids [6, 7]. It can be performed preoperatively or postoperatively or can also be given alone. Postoperative radiotherapy has been demonstrated to be able to reduce the recurrence of keloids and to be safe [8, 9]. A relatively novel photon therapy apparatus, Intrabeam, has been used in the treatment of breast cancer [10], brain cancer [11], rectal cancer [12], and vertebral metastases [13, 14] because of its small size, light weight, ease of transportation, and low operating room protection requirements. As the Intrabeam system (Zeiss Corporation) uses low-energy X-rays, patients require less protection and sustain minimal damage to tissue surrounding the target area. The system is therefore ideal for treating superficial lesions like keloids. In this study, we assessed its efficacy in patients with keloids.

## 2. Materials and Methods

Fourteen patients with keloids underwent radiotherapy using the Intrabeam system from November 2016 to March 2018. We also compared our data from this cohort to earlier data from keloid patients who had previously been exposed to 6 MV electron beams using conventional accelerators from January 2015 to December 2016. The Intrabeam system (Zeiss Corporation) uses low-energy X-rays. And the 6 MV electron beams group patients were delivered using Siemens Oncor linac. Our inclusion criteria were (i) pathological diagnoses, and (ii) the patient agrees to this treatment. The exclusion criteria were (i) pregnancy and lactation, (ii) contraindications to radiation therapy, (iii) incomplete data, and (iv) lack of willingness to participate. We obtained informed consent from all patients. This work was approved by the hospital's ethics committee.

The follow-up period ranged from 3 to 32 months in the two groups. During the follow-up, patients were asked to have an outpatient visit at 1 month, 3 months, and 6 months and annually after treatment. Recurrence is defined as pain, pruritus from the scars, clinically visible a mass, or significant recurrence of the lesion. Our technical staffs recorded these details following a strict protocol.

## 3. Results

**Figure 1:**
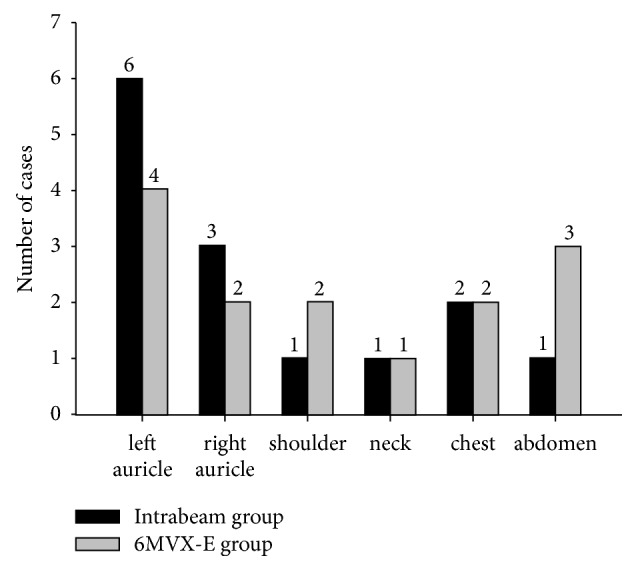
The frequency of keloid lesions in different areas.

**Figure 2:**
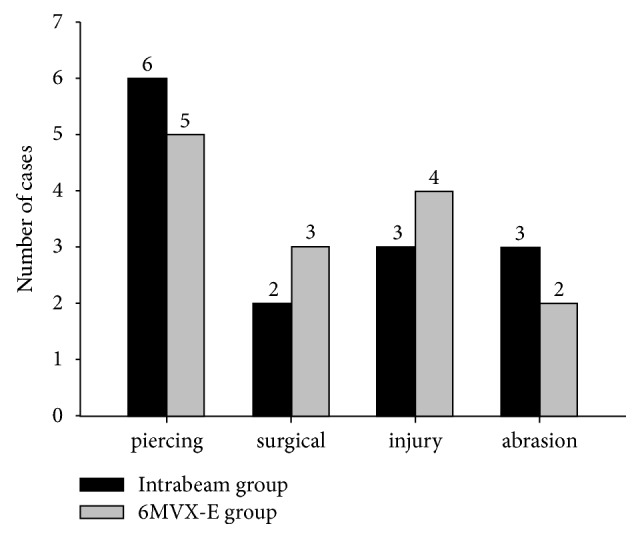
The etiology of the keloid lesions.

The Intrabeam group has a total of 12 females and 2 males, with an average age of 38 years old (range: 21–60 years). One patient had two keloids, on both left and right ear auricles. The other 13 cases had only one keloid each, located on the auricle, neck, shoulder, chest, or abdomen (Figure 1). The causes of the keloid lesions include piercings (6 cases), injury (3 cases), surgical trauma (2 cases), and abrasion scars (3 cases) (Figure 2). Two of the 14 patients had relapsed keloids after previous surgery. One of these two had a keloid that could not be surgically removed because it was too large. The other twelve all had newly diagnosed keloids. The patient who could not be operated upon underwent 5 Gy of Intrabeam radiation therapies twice. Its reference depth was 0.5 mm below the skin. The other 13 patients first underwent surgical resection of keloids. Two radiotherapy sessions were then performed at 0 and 3 days after surgery. The dose was 4 or 5 Gy each time and the duration of radiation therapy ranged from 3 min 14 s to 12 min 01 s. The reference depth is 0 mm. The total dose at 0mm is 4.0Gy-11.8Gy, and dose at 5mm is 1.7Gy-5.0Gy. The clinical data of all patients and the details about the technical delivery of Intrabeam beams are displayed in Table 1.

The 6 MV electron beam group has 10 female patients and 4 male patients, with an average age of 41 years (range: 19–74 years). The sites and etiologies of the keloid are shown in Figures 1 and 2. The keloids of these patients were first surgically removed, followed by three radiotherapy sessions 0, 2, and 4 days after surgery. The beam energy is 6MeV. Each dose at 0mm and 5mm was 4 Gy. The details about the clinical data and the technical delivery of electron beams are shown in Table 2.

**Figure 3:**
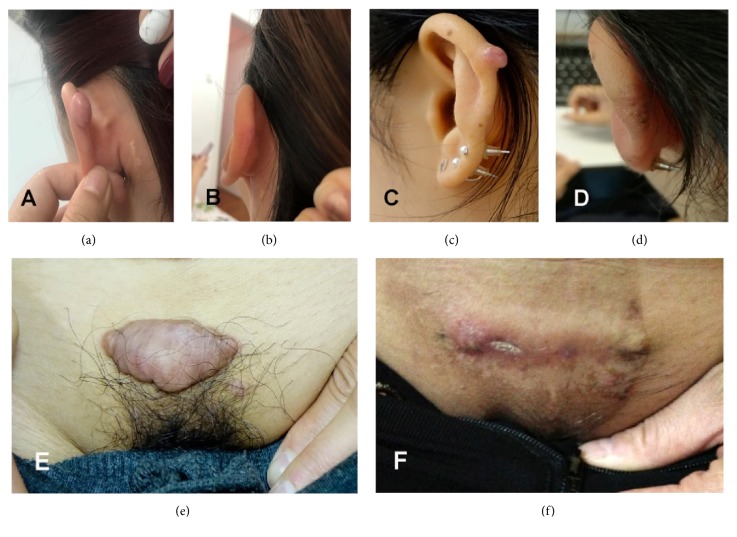
Before surgical resection and after radiotherapy ((a-b): Patient 1; (c-d): Patient 2; (e-f): Patient 3).

**Figure 4:**
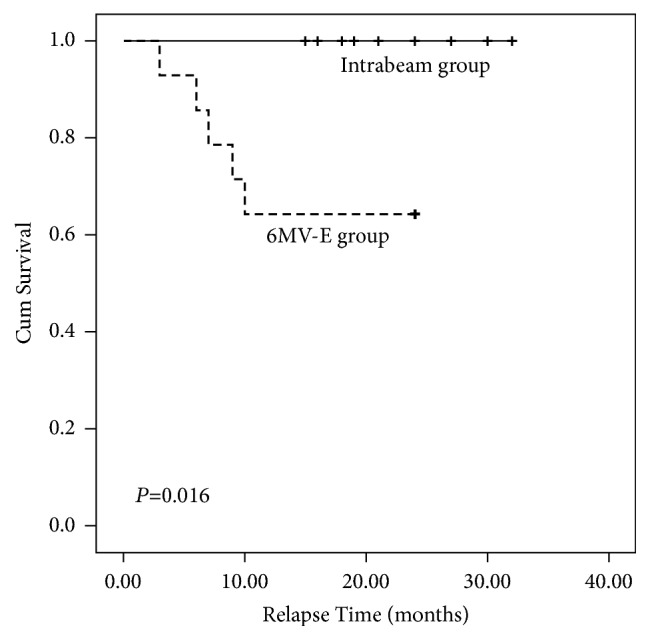
The average time to relapse.

The follow-up period ranged from 15 to 32 months (median: 22.5 months) in Intrabeam group and 3–24 months (median: 24 months) in 6MV-E group. We collected the photos of patients before surgery and before and after radiotherapy (Figure 3). The treatment showed beneficial effects on the patients' appearance, quality of life, and self-confidence. In the Intrabeam group, only one patient, who could not undergo surgical resection after recurrence due to size, reported adverse reactions—superficial ulcers appeared one month after radiotherapy. This patient had two doses of 5 Gy of radiation therapy and the reference depth was 0.5 mm below the skin. Because of the unique condition, the irradiation dose on the epidermis is much higher than 5 Gy, so it is not unreasonable to see a higher possibility of ulcers. The other 13 patients in the Intrabeam group were postoperative, and their prescription reference dose depth was 0mm at the skin. The skin of a part of the patients' treatment areas showed mild hyperpigmentation within one month which spontaneously resolved after one more month without blisters, flaky peeling, or any other symptoms. A small percentage of patients experienced mild, short-term skin itching. The zero recurrence rate of Intrabeam group was significantly lower than in the group exposed to 6 MV electron beam irradiation (*P*=0.016, Figure 4). Excellent cosmetic results were reported in 90% of the Intrabeam group patients.

## 4. Discussion

Skin keloids are scars with severe fibrous tissue and vitreous degeneration around the wound after skin damage or surgery. They are hard, elastic nodules or plaques that are formed by the hyperplasia of connective tissues. Proliferative skin keloids are a result of hypoxia [15]. Hypoxia causes capillaries to dilate, resulting in the proliferation and differentiation of endothelial cells into myofibroblasts and fibroblasts. These cells cause a large amount of collagen synthesis and fibrin deposition, which could lead to increased blood vessel blockage, further hypoxia, and proliferative skin scar formation [15]. Keloids grow faster than the skin tissue. They extend beyond the original sites of the lesion to nearby features. The disease is common in adolescents and young women [16], with a variety of causes. Keloids are mostly purple, often accompanied by obvious pain or itching, affecting the patients' work and rest. The condition often relapses after surgery. Relapsed keloids grow even faster than those original ones before surgery and the affected areas tend to be larger.

The electron beam is used clinically because of radiation's many characteristics. Radiation has a beneficial effect on superficial tumors on the surface of the skin. From the surface of the skin to a certain depth, its dose distribution is even [17–19]. The Intrabeam system uses low-energy X-rays that attenuate rapidly. Tissues and vital organs are therefore protected from radiation. Intrabeam technology progressed rapidly in recent years and is widely used in the treatment of breast cancer [20], brain tumor [11], metastatic spinal cord cancer [21], and rectal cancer [12]. Its advantages are as follows: (i) The treatment time is short, which greatly shortens the interval between surgery and other treatment methods and overall treatment time. (ii) Through accurate target positioning, we can better protect the surrounding normal tissue and improve local irradiation. (iii) The more uniform dose coverage and fewer adverse reactions allow better postoperative cosmetic results. (iv) The local recurrence rate in the treatment of breast and colorectal cancer is low. (v) Small size, light weight, ease of transportation, low energy, and low operating room protection requirements are there. All these have allowed the Intrabeam system to come into general use in the operating room.

Several methods are available for postoperative radiotherapy of keloids. At present, most commonly used ones are superficial X-ray [22], electron ray external radiation [23], and ^32^P-patch irradiation [24]. It is generally believed that the mechanism of radiation therapy involves radiation energy inhibiting fibrosis in the scar tissue [3]. Ideally, a 24-h interval between surgery and radiotherapy is best for electronic beam treatment. During wound healing after surgery, balancing of collagen formation and degradation is disturbed or destroyed, resulting in the accumulation of collagen fibers and the formation of a large number of collagen fibrils, rendering the anabolism of collagen greater than catabolism [25]. Immature fibroblasts occupy most of the incisions within 24 h of surgery [26]. Unstable collagen fibers from these cells are the main component and are more sensitive to radiation than other fibers. Radiation can effectively inhibit the proliferation of fibroblasts, inhibit the growth of capillary sprouts at the incision, reduce the amount of inflammation, push the collagen fiber metabolism toward a relative balance, and also have a certain degree of hemostasis and anti-infection ability [27].

Reported rates of keloid recurrence vary a lot. According to Ahmadreza's data, the rate of recurrence during 2 years of follow-up was 20%. Patients in general experience aesthetic improvement and clinical symptomatic relief (pruritus, pain, and other complaints) and have a degree of satisfaction of more than 80% [28]. However according to some other studies on simple surgical resection, relapse rate exceeded 80% [29, 30]. For brachytherapy after surgery, one study followed up 35 patients with keloids, and only 1 of them had recurrence [31]. In another similar study, 21 patients with keloids were evaluated for early results of brachytherapy after surgery, and 2 had local recurrence [32], indicating that brachytherapy was effective after surgery in reducing recurrence.

We performed two rounds of Intrabeam radiotherapy within 3 days of surgery, which was well tolerated. Our patients had no serious complications after radiotherapy, and no one complained of pain or any other discomfort. The advantages of our program are good cosmetic results, low complication rate, low recurrence rate, and better long-term treatment effect. Since our radiation therapy involves a split dose of 4–5 Gy in each treatment regimen, the 0 recurrence rate can be brought down to the levels seen in other studies. Flickinger [7] found a strong dependence between local control and dose and estimated at least 16Gy or 22Gy in 3 fractions was needed for 90% control of earlobe and nonearlobe sites, respectively. And, Kovalic and colleagues [30] analyzed results of 107 patients with keloids were treated with radiation therapy at the Mallinckrodt Institute of Radiology. Of these, most of the patients were treated with 12 Gy in three fractions of 4 Gy over 3 days. There were no complications from this low dose treatment. Their results prove that their treatment and therapeutic dose are effective and safe for keloids. In our treatment, the dose is 8-10Gy in 2 fractions in Intrabeam group and 12Gy in 3 fractions the electron group. Our treatment is equally effective and safe. High doses of radiation pose a safety hazard to the human body. Since the same effect can be achieved, we prefer a low dose method. Overall, the present work and other studies showed surgical resection combined with radiotherapy to have a pronounced advantage in the treatment of keloids, including painlessness, minimal expense, and ease. Our radiotherapy regimen can be performed in outpatients without serious contraindications, with high local control rate, good patient tolerance, reasonable radiation dose distribution, and low exposure dose to normal tissue.

Our current study has some flaws. First, the number of patients selected was small. Second, the patients' conditions were complicated. Some patients had recurrence and could not be operated upon again. We hope that in future studies, the number of patients would be increased and longer-term follow-ups can be performed to observe the efficacy.

All in all, we recommend using the Intrabeam system to treat keloids. However, further studies are needed to support this conclusion.

## Figures and Tables

**Table 1:** Clinical parameters of patients treated with Intrabeam system.

Case	Gender	Age(year)	XRS(KV)	Dose at 0mm(Gy)	Dose at 5mm(Gy)	Applicator type	Applicator Size(cm)	Treatment time	Follow up (mo)
1	Female	60	50	11.8	5.0	flat	3.0	9:03+9:04	32
2	Female	45	50	5.0	2.1	flat	3.0	3:17+3:17	30
3	Female	20	50	4.0	1.7	flat	3.0	3:16+3:17	30
4	Female	21	50	5.0	2.1	flat	3.0	4:07+4:04	27
			50	5.0	2.1	flat	3.0	4:06+4:06	27
5	Female	26	50	5.0	2.4	flat	4.0	6:53+4:04	24
6	Female	23	50	4.0	1.7	flat	3.0	3:14+3:15	24
7	Female	23	50	5.0	2.1	flat	3.0	3:54+3:58	21
8	Female	22	50	5.0	2.1	flat	3.0	4:21+4:20	19
9	Female	24	50	4.0	2.1	flat	5.0	7:50+7:49	18
10	Female	22	50	5.0	2.1	flat	3.0	3:28+3:29	16
11	Male	48	50	5.0	2.7	flat	6.0	11:59+12:00	16
12	Female	28	50	5.0	2.7	flat	6.0	12:01+11:50	15
13	Male	22	50	5.0	2.6	flat	5.0	9:43+9:43	15
14	Female	23	50	5.0	2.6	flat	5.0	9:45+9:45	15

**Table 2:** Clinical parameters of patients treated with electron beams.

Case	Gender	Age(year)	beam energy(MeV)	Blous (cm)	Field size (cm*∗*cm)	Dose at 0mm(Gy)	Dose at 5mm(Gy)	Follow up (mo)
1	Female	31	6	1	4*∗*3	4.0	4.0	10
2	Female	21	6	1	5*∗*3	4.0	4.0	3
3	Male	57	6	1	7*∗*5	4.0	4.0	9
4	Male	35	6	1	4*∗*4	4.0	4.0	6
5	Female	60	6	1	10*∗*6	4.0	4.0	24
6	Female	37	6	1	6*∗*7	4.0	4.0	24
7	Male	19	6	1	5*∗*5	4.0	4.0	24
8	Female	65	6	1	9*∗*6	4.0	4.0	24
9	Female	25	6	1	4*∗*5	4.0	4.0	24
10	Female	40	6	1	4*∗*3	4.0	4.0	24
11	Female	27	6	1	4*∗*4	4.0	4.0	24
12	Female	74	6	1	4*∗*3	4.0	4.0	24
13	Female	27	6	1	4*∗*4	4.0	4.0	24
14	Male	56	6	1	4*∗*5	4.0	4.0	7

## Data Availability

The data used to support the findings of this study are available from the corresponding author upon request.

## Supplementary Materials

Supplementary Materials Table s1 The details on the technical delivery of Intrabeam. Table s2 The details on the technical delivery of electron beams.
