# Supplementary material for: A Novel Radiotherapy Approach for Keloids with Intrabeam
Source: Biomed Res Int. 2019 Jul 22;2019:4693528. doi: 10.1155/2019/4693528 (PMC6679878; doi:10.1155/2019/4693528)
Supplement: Supplementary Materials — Table s1 The details on the technical delivery of Intrabeam. Table s2 The details on the technical delivery of electron beams. [file 4693528.f1.docx]

| Table s1 The details on the technical delivery of Intrabeam | | | | | | |
| --- | --- | --- | --- | --- | --- | --- |
| Case | Dose at 0mm | Dose at 5mm | XRS | Applicator  type | Applicator  Size(cm) | Applicator  serial number |
| 1 | 11.82Gy | 5Gy | 50KV | flat | 3.0 | 121002 |
| 2 | 5Gy | 2.115Gy | 50KV | flat | 3.0 | 121002 |
| 3 | 4Gy | 1.692Gy | 50KV | flat | 3.0 | 121002 |
| 4 | 5Gy | 2.115Gy | 50KV | flat | 3.0 | 121002 |
|  | 5Gy | 2.115Gy | 50KV | flat | 3.0 | 121002 |
| 5 | 5Gy | 2.435Gy | 50KV | flat | 4.0 | 121002 |
| 6 | 4Gy | 1.692Gy | 50KV | flat | 3.0 | 121002 |
| 7 | 5Gy | 2.115Gy | 50KV | flat | 3.0 | 121002 |
| 8 | 5Gy | 2.115Gy | 50KV | flat | 3.0 | 121002 |
| 9 | 4Gy | 2.088Gy | 50KV | flat | 5.0 | 141037 |
| 10 | 5Gy | 2.115Gy | 50KV | flat | 3.0 | 121002 |
| 11 | 5Gy | 2.72Gy | 50KV | flat | 6.0 | 151001 |
| 12 | 5Gy | 2.72Gy | 50KV | flat | 6.0 | 151001 |
| 13 | 5Gy | 2.61Gy | 50KV | flat | 5.0 | 141037 |
| 14 | 5Gy | 2.61Gy | 50KV | flat | 5.0 | 141037 |

| Table s2 The details on the technical delivery of electron beams | | | | | |
| --- | --- | --- | --- | --- | --- |
| Case | Linac type | Blous | Field size (cm*cm) | Dose at 0mm | Dose at 5mm |
| 1 | ONCOR; Siemens | 1cm | 4*3 | 4Gy | 4Gy |
| 2 | ONCOR; Siemens | 1cm | 5*3 | 4Gy | 4Gy |
| 3 | ONCOR; Siemens | 1cm | 7*5 | 4Gy | 4Gy |
| 4 | ONCOR; Siemens | 1cm | 4*4 | 4Gy | 4Gy |
| 5 | ONCOR; Siemens | 1cm | 10*6 | 4Gy | 4Gy |
| 6 | ONCOR; Siemens | 1cm | 6*7 | 4Gy | 4Gy |
| 7 | ONCOR; Siemens | 1cm | 5*5 | 4Gy | 4Gy |
| 8 | ONCOR; Siemens | 1cm | 9*6 | 4Gy | 4Gy |
| 9 | ONCOR; Siemens | 1cm | 4*5 | 4Gy | 4Gy |
| 10 | ONCOR; Siemens | 1cm | 4*3 | 4Gy | 4Gy |
| 11 | ONCOR; Siemens | 1cm | 4*4 | 4Gy | 4Gy |
| 12 | ONCOR; Siemens | 1cm | 4*3 | 4Gy | 4Gy |
| 13 | ONCOR; Siemens | 1cm | 4*4 | 4Gy | 4Gy |
| 14 | ONCOR; Siemens | 1cm | 4*5 | 4Gy | 4Gy |
